# Apaf-1/caspase-4 pyroptosome: a mediator of mitochondrial permeability transition-triggered pyroptosis

**DOI:** 10.1038/s41392-021-00524-4

**Published:** 2021-03-09

**Authors:** Gang Shao, Lingfei Wang, Xi Wang, Caiyun Fu

**Affiliations:** 1 Department of Oncology, No. 903 Hospital of PLA Joint Logistic Support Force, Xi Hu Affiliated Hospital of Hangzhou Medical College, Hangzhou, China; 2 Zhejiang Provincial Key Laboratory of Silkworm Bioreactor and Biomedicine, College of Life Sciences and Medicine, Zhejiang Sci-Tech University, Hangzhou, China

**Keywords:** Molecular medicine, Diseases

In a recent paper in *Cell Metabolism*, Xu et al. provide new insights whether and how caspase-4/11 involves in pyroptotic cell death implicated in noninfective diseases. They discovered a novel mechanism of GSDME-dependent pyroptosis, which was induced by mitochondrial permeability transition (MPT)-activated Apaf-1/caspase-4 pyroptosome assembly.^1^ These findings provide important implications for understanding the pathogenesis of cholestatic liver failure (Fig. 1).

**Fig. 1:**
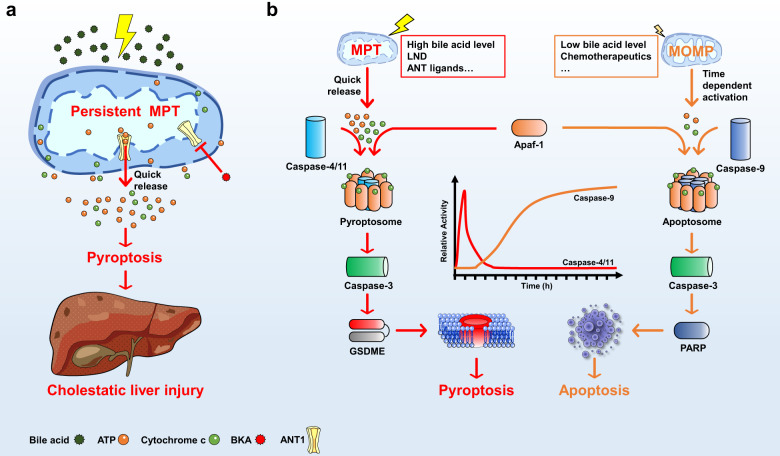
Bile acids activate MPT-triggered pyroptosis by Apaf-1 pyroptosome and cause cholestatic liver injury. **a** Bile acids of high level activate persistent MPT to quickly release ATP and cytochrome c through channels such as ANT1 to promote pyroptosis and cause cholestatic liver injury. **b** A novel molecular mechanism that Apaf-1 senses MPT to activate caspase-4/11-caspase-3-GSDME signal axis to promote pyroptosis, while the apoptosis pathway was activated by MOMP stimulation

Pyroptosis is one type of programmed cell death with the features of pore formation on the plasma membrane, cell swelling and lysis. Growing evidences indicated that GSDMD-mediated pyroptosis plays a key role in lots of diseases, such as microbial infection, septic shock, inflammation in noninfectious conditions, sterile inflammation in liver disease, experimental autoimmune encephalomyelitis in mice, and other inflammation diseases associated with NLRP3 and IL-1β.^2^ In 2017, Emad S. Alnemri’s laboratory and Feng Shao’s laboratory successively found that chemotherapy can transform apoptosis to pyroptosis by activating caspase-3 cleavage of GSDME,^3,4^ initially revealing the molecular mechanism of how GSDME involved in pyroptosis. Caspase-4 (homolog of mouse caspase-11) is a non-canonical inflammasome detected cytoplasmic LPS to active GSDMD and involved in pyroptotic cell death implicated in infectious diseases,^5^ but it is unclear whether and how caspase-4 senses host-derived endogenous factors to trigger pyroptosis in noninfective diseases.

In this research, the authors firstly detected whether bile acids (DCA and CDCA) have the ability to trigger pyroptotic cell death. They showed that bile acid-induced cell death was blocked by neither RIP1 inhibitor nor MLKL inhibitor. Further, bile acid-induced cell death was recovered in mouse bone marrow-derived macrophages (BMDMs) isolated from *Caspase-11*^*−/−*^ mice, indicating that bile acids triggered caspase-4/11-dependent pyroptosis, instead of programmed necroptosis. Unexpectedly, bile acids inhibited the binding between caspase-11 and GSDMD but promoted the cleavage of caspase-3/GSDME, which suggests that bile acids may activate a novel pyroptosis signal axis, namely caspase-4/11-caspase-3-GSDME axis. It is an attractive finding that caspase-4/11, a novel identified upstream molecular signal of caspase-3, mediates pyroptosis through the cleavage of GSDME.

So the question is coming that how caspase-4 is activated in the stimulation of bile acids to trigger pyroptosis? The result of microscale thermophoresis analysis demonstrated that bile acids were unable to directly bind with caspase-4/11. To further explore certain cell-intrinsic factors to mediate the activation of caspase-4, a panel of inhibitors for mitochondrial permeability transition pore (MPTP) were screened and found that an adenine nucleotide translocator (ANT) inhibitor bongkrekic acid (BKA) could significantly protect cell death and inhibit bile acid-induced MPT and pyroptosis signals. The authors then used thapsigargin (TG) and lonidamine (LND) to induce MPT, confirming that caspase-4/11 is a general sensor of MPT.

In order to further clarify the signal transfer factors downstream of MPT to activate caspase-4, protein interaction techniques were used to identify the proteins that interacted with caspase-4. The authors surprisingly identified Apaf-1, the key adaptor of intrinsic apoptosis, interacted with caspase-4 directly, and they named this protein complex as Apaf-1 pyroptosome. In order to verify the Apaf-1 pyroptosome was sufficient and necessary in MPT-induced pyroptosis, *APAF-1* knockout HepG2 cells were used with the results that bile acids were failed to activate caspase-4-caspase-3-GSDME signaling. Furthermore, ATP and cytochrome c were verified as cofactors for Apaf-1/caspase-4 complex to activate pyroptosis. In addition, the activated caspase-4 cleaved caspase-3 instead of GSDMD. All these results proved that Apaf-1/caspase-4 pyroptosome assembly was an upstream event of the caspase-3-GSDME signal axis. These interesting findings uncover different signal axes of caspase-4 to activate GSDMD or GSDME depending on the combination with LPS or Apaf-1, respectively. In particular, it is still unclear whether Apaf-1/caspase-4 is a general signal for activating caspase-3-GSDME. More and more cumulative evidences have proved that Apaf-1 plays important roles in apoptosis signaling pathway. So it is necessary to verify the relationship between Apaf-1 and pyroptosis in the near future.

Next, the authors needed to uncover the critical molecular mechanisms to dictate apoptosome and pyroptosome assembly under mitochondrial stress. They performed a time-course study of caspase activation and found that low-dose bile acids promoted mitochondrial outer membrane permeabilization (MOMP) and activated caspase-9 after a longer period of time, while high-dose bile acids induced MPT and quickly activated caspase-11. Further research found that MPT could release cytochrome c and ATP more rapidly than MOMP, and the molar ratio of Apaf-1 to caspase is 7:2 in pyroptosome instead of 7:4 in apoptosome. These results suggested that the stronger stimulation and a larger stoichiometric ratio of Apaf-1 to caspase may be the reason why Apaf-1 selectively assembles pyroptosome. It is still unclear whether there is an intermediate state in which a certain stimulation concentration of bile acid is insufficient to trigger complete pyroptosis but co-activate both pyroptosis and apoptosis. Mitochondria are closely related to apoptosis, necroptosis, and pyroptosis. Understanding the dynamic changes and molecular activities of mitochondria may be a key pointcut in the process of cell death. So it is very important to clarify the molecular events to mediate apoptosis, necroptosis, and pyroptosis in response to mitochondrial stress in the future.

Finally, the authors wanted to ask whether Apaf-1/caspase-4 pyroptosome signal axis triggered by MPT is pathophysiologically relevant. Thus, a cholestasis model by bile duct ligation (BDL) was constructed to form acute liver injury with an accumulation of bile acids in the circulating system. The results showed that BDL mice had persistent MPT characterized by loss of mitochondria cristae. Furthermore, there was activation of Apaf-1/caspase-11 pyroptosome and caspase-3-GSDME signal axis in liver tissue. *Caspase-11*^−/−^ BDL mice inhibited the pyroptotic signal axis, and *GSDME*^−/−^ mice almost completely alleviated the death caused by BDL. These results indicated that the pyroptosis induced by caspase-4/11-caspase-3-GSDME axis is the dominant factor of cholestatic liver failure.

Taken together, Xu et al. uncovered a novel mechanism of pyroptosis under sterile conditions, in which Apaf-1 played a key role in sensing different extents/types of stimuli of mitochondrial stress and in the selection of pyroptosis or apoptosis. In the near future, scientists will pay more attention to uncover the exact molecular mechanisms underlying Apaf-1 differentially recruited caspase-9 and caspase-4 under different conditions. In a word, this research clarified a novel molecular event of MPT-driven cell death, and provided a new direction for the treatment of MPT-driven diseases such as cholestatic liver failure.

## References

[1] Xu W (2021). Apaf-1 pyroptosome senses mitochondrial permeability transition. Cell Metab..

[2] Orning P (2019). Gasdermins and their role in immunity and inflammation. J. Exp. Med..

[3] Wang Y (2017). Chemotherapy drugs induce pyroptosis through caspase-3 cleavage of a gasdermin. Nature.

[4] Rogers C (2017). Cleavage of DFNA5 by caspase-3 during apoptosis mediates progression to secondary necrotic/pyroptotic cell death. Nat. Commun..

[5] Wang K (2020). Structural mechanism for GSDMD targeting by autoprocessed caspases in pyroptosis. Cell.

